# Supplementary material for: Sea Cucumber (Isostichopus badionotus): Bioactivity and Wound Healing Capacity In Vitro of Small Peptide Isolates from Digests of Whole-Body Wall or Purified Collagen
Source: Mar Drugs. 2025 Oct 22;23(11):411. doi: 10.3390/md23110411 (PMC12653620; doi:10.3390/md23110411)
Supplement: Supplementary file 1 [file marinedrugs-23-00411-s001.zip › marinedrugs-3899334-supplementary.pdf]

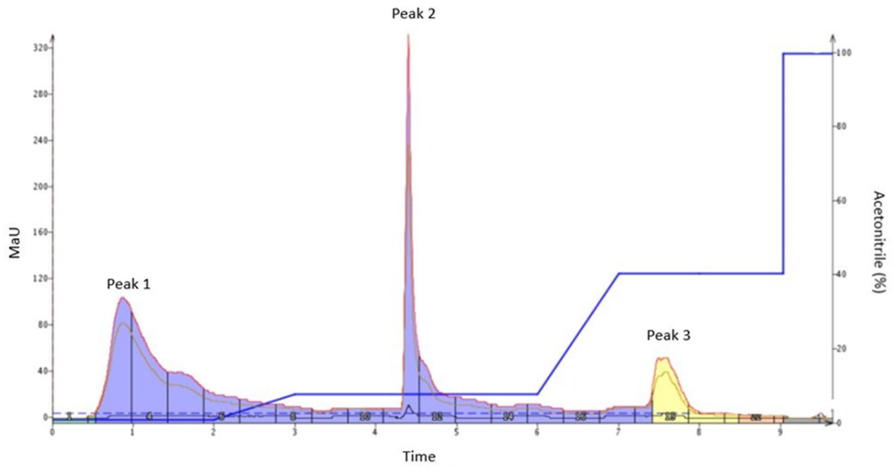

Supplemental Figure S1. Separation of the *I. badionotus* collagen 1-3 kDa fraction by flash chromatography (Original printout).

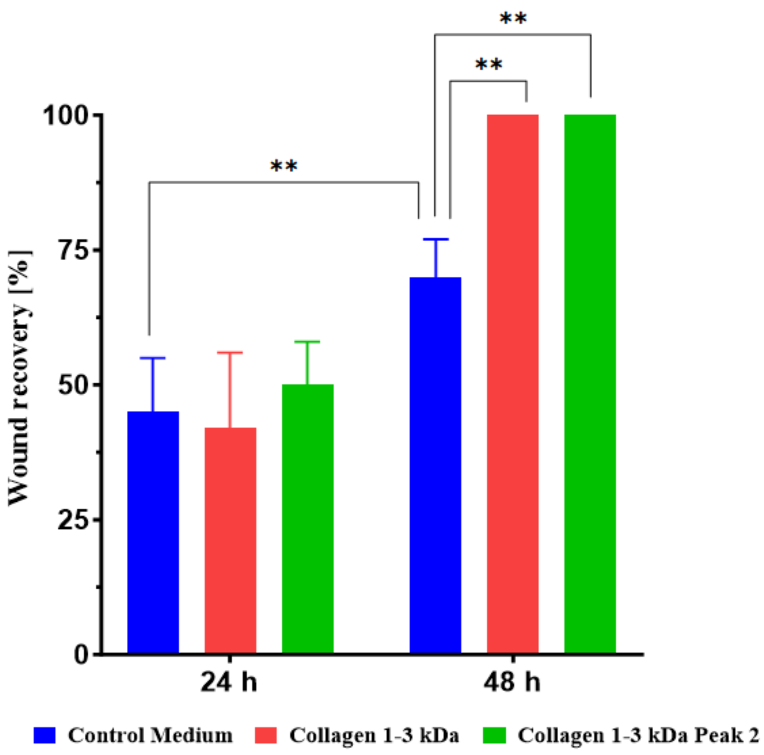

Supplemental Figure S2. Evaluation of wound healing in a scratch assay based on human keratinocytes cultured in medium containing 1% foetal calf serum plus or minus, Collagen 1-3 kDa or Collagen 1-3 kDa Peak 2 for 24 h and 48 h. N=3 and ** indicates statistical significance ($p \leq 0.01$).

Supplemental Table S1. Comparison of *I. badionotus* collagen parameters (present study) with those published for several other sea cucumber species.

Species	Yield (% DW)	α-chain (kDa)	Td (°C)	Imino acids (%)	Gly (%)	Ala (%)
S. japonicas	26.6	135	35.3	19.2	32.5	9.8
H. Scabra	8.2	110-130	32.3	18.5	18.2	10.5
I. badionotus	16.5	131	32.5	17.2	28.9	11.0
H. nobilis	28.8	80-90	34.6	16.8	28.0	11.0
S. vastus	21.3	122	21.3	16.4	32.2	10.8
S. horrens	33	125	30.0	16.1	32.5	11.0
A. leucoprocta	44.0	110-130	25.4	16.0	30.0	9.0
H. arenicola	17.0	125	34.6	15.9	32.5	11.3
H. cinerascens	72.2	80-90	30.0	15.8	31.0	11.0
H. parva	7.0	130	32.5	15.8	27.0	9.1
H. leucospilota	27-30	133-166	34.6	15.8	43.8	2.2
S. monotuberculatus	2.6	137	30.2	15.1	32.0	9.2
P. californicus	20.0	138	17.9	14.2	32.5	11.2
A. mollis	10.0	116	46.7	8.8	28.6	15.9

References: [4][6][20][32][40][42-43][48] [95-105]
